# Random Walks and Lorentz Processes

**DOI:** 10.3390/e26110908

**Published:** 2024-10-25

**Authors:** Domokos Szász

**Affiliations:** Department of Stochastics, Budapest University of Technology and Economics, H-1111 Budapest, Hungary; szasz@math.bme.hu

**Keywords:** random walk, Lorentz process, recurrence

## Abstract

Random walks and Lorentz processes serve as fundamental models for Brownian motion. The study of random walks is a favorite object of probability theory, whereas that of Lorentz processes belongs to the theory of hyperbolic dynamical systems. Here we first present an example where the method based on the probabilistic approach led to new results for the Lorentz process: concretely, the recurrence of the planar periodic Lorentz process with a finite horizon. Afterwards, an unsolved problem—related to a 1981 question of Sinai on locally perturbed periodic Lorentz processes—is formulated as an analogous problem in the language of random walks.

## 1. Introduction

According to Pólya’s classical theorem [1], the simple symmetric random walk (SSRW) on $Z^{d}$ is recurrent if d=1,2; otherwise, it is transient. Since the one- and two-dimensional Lorentz processes with a periodic configuration of scatterers share a number of stochastic properties with those of SSRWs, it had also been expected that the analogues of Pólya’s theorem also hold for them. Indeed, in d=2, the recurrence of the periodic finite-horizon Lorentz process (FHLP) was settled by ergodic theoretic methods independently by K. Schmidt [2] and J.-P. Conze [3]. For the same case, in 2004, with T. Varjú, we succeeded in giving a probabilistic–dynamical proof in [4]. The recurrence of the infinite-horizon Lorentz process (IHLP) in the plane was open until 2007, when the method of [4] could be extended to the infinite-horizon case (see [5]). It is worth mentioning that in the infinite-horizon case, the limit law of the Lorentz process belongs to the non-standard domain of attraction of the normal law, in contrast with the finite-horizon case where convergence to the normal law (and to the Wiener process) holds with the diffusive scaling.

Sinai asked in 1981 whether probabilistic properties of the Lorentz process remain valid if one changes the scatterer configuration in a bounded domain. For convergence to the Gaussian law (and to the Wiener process) and, moreover, for the local limit theorem (LLT) and for recurrence, we gave an affirmative answer in [6]. It is worth mentioning that—in our probabilistic approach and for our purposes solely—the recurrence property can be positioned above the limit theorem (with diffusive or over-diffusive scaling) and even over the LLT, see Section 5. Of course, other properties are also very important, such as the speed of correlation decay, almost sure invariance principle, etc. (cf. [7]).

However, as to Sinai’s 1981 question, nothing is known in the infinite-horizon case. Since the study of random walks might be much instructive for the behavior of Lorentz processes—in Section 5—we formulate some problems for random walks with unbounded jumps that are most interesting in themselves. Before the closing section, this paper contains a survey of results known for recurrence properties of RWs and of Lorentz processes.

## 2. Random Walks and Lorentz Processes

### 2.1. Random Walks

**Definition** **1** (random walk)**.**

*1.*  *Let $\left\{X_{n}\in Z^{d}|n\geq 0\right\}$ be independent random variables, and for n≥0, denote*
$$S_{n}=\sum_{j=1}^{n}X_{j}.$$
  *Then the Markov chain $S_{0},S_{1},S_{2},\ldots ,S_{n},\ldots$ is called a* random walk.
*2.*  *The random walk is called* symmetric *if the $X_{n}$s are symmetric random variables.*
*3.*  *The random walk is* simple *if $|X_{n}|=1\;\mathrm{holds}\;\forall n\geq 0.$*
*4.*  *The probabilities*
$$P(X_{n+1}=k|S_{n})$$
  *are called the* transition (or jump) probabilities *of the random walk.*
*5.*  *The random walk is called* translation-invariant *(or classical or homogeneous) if its transition probabilities are translation-invariant.*

As to basic notions and properties of random walks, we refer to the monographs [8,9,10].

**Definition** **2** (locally perturbed random walk)**.** *Assume a>0. If—possibly outside an origo-centered cube $Q_{a}$ of size a—the translation probabilities of a random walk $S_{0},S_{1},S_{2},\ldots ,S_{n},\ldots$ are translation-invariant, then we say that the random walk is a* locally perturbed random walk *(LPRW) (more precisely an a*-locally perturbed random walk*). For simplicity, we assume that all transition probabilities are bounded away from 0.*

### 2.2. Sinai Billiards and Lorentz Processes

#### 2.2.1. Sinai Billiards

As far as notations go, we mainly follow [11] for planar billiards and [12] for multidimensional ones.

Billiards are defined in Euclidean domains bounded by a finite number of smooth boundary pieces. For our purpose, a *billiard* is a dynamical system describing the motion of a point particle in a connected, compact domain $Q\subset T^{d}=R^{d}/Z^{d}$. In general, the boundary ∂Q of the domain is assumed to be piecewise $C^{3}$-smooth, i.e., there are no corner points; if 0<J<∞ is the number of such pieces, we can write $\partial Q=\cup_{1\leq \alpha \leq J}\;\partial Q_{\alpha }$. Connected components of $T^{d}\setminus Q$ are called *scatterers* and are assumed to be strictly convex. Motion is uniform inside Q, and specular reflections take place at the boundary ∂Q; in other words, a particle propagates freely until it collides with a scatterer, where it is reflected elastically, i.e., following the classical rule that the angle of incidence be equal to the angle of reflection.

**Definition** **3** (Sinai billiard)**.** *A billiard with strictly convex scatterers is called a* Sinai billiard.

**Remark** **1.** *The above notion of a Sinai billiard is more general than its original one where it was supposed that d=2,J=1, and $Q_{1}$ was a circle.*

Since the absolute value of the velocity is a first integral of motion, the phase space of our billiard is defined as the product of the set of spatial configurations by the (d−1)-sphere, $M=Q\times S_{d-1}$, which is to say that every phase point x∈M is of the form x=(q,v), with q∈Q and $v\in R^{d}$ with norm |v|=1. According to the reflection rule, M is subject to identification of incoming and outgoing phase points at the boundary $\partial M=\partial Q\times S_{d-1}$. The billiard dynamics on M is called the *billiard flow* and denoted by $S^{t}:t\in \left(-\infty ,\infty \right)$, where $S^{t}:M\to M$. The set of points defined by the trajectory going through x∈M is denoted as $S^{R}x$. The smooth, invariant probability measure of the billiard flow, μ on M, also called the Liouville measure, is essentially the product of Lebesgue measures on the respective spaces, i.e., dμ=const.dqdv, where the constant is ${\left(\mathrm{vol}\;Q\;\mathrm{vol}\;S_{d-1}\right)}^{-1}$.

The appearance of collision–free orbits is a distinctive feature of some billiards which are said to have infinite horizons.

**Definition** **4** (infinite and finite horizons)**.**

*1.*  *Denote by $M_{\mathrm{free}}\subset M$ the subset of collision-free orbits, i.e.,*
$$M_{\mathrm{free}}=\left\{x\in M\;:\;S^{R}x\cap \partial M=\emptyset \right\}\;.$$
*2.*  *The billiard has a* finite horizon *if $M_{\mathrm{free}}=\emptyset$. Otherwise it has an* infinite horizon.

#### 2.2.2. Lorentz Processes

The Lorentz process was introduced in 1905 by H. A. Lorentz [13] for the study of a dilute electron gas in a metal. While Lorentz considered the motion of a collection of independent pointlike particles moving uniformly among immovable metallic ions modeled by elastic spheres, we consider here the uniform motion of a single pointlike particle in a fixed array of strictly convex scatterers with which it interacts via elastic collisions.

Thus defined, the *Lorentz process* is the billiard dynamics of a point particle on a billiard table $Q=R^{d}\setminus \cup_{\alpha =1}^{\infty }\;O_{\alpha }$, where the scatterers $O_{\alpha }$, 1≤α≤∞, are strictly convex with $C^{3}$-smooth boundaries. Generally speaking, it could happen that Q has several connected components. For simplicity, however, we assume that the scatterers are disjoint and that Q is unbounded and connected. The phase space of this process is then given according to the above definition, namely, $M=Q\times S_{d-1}$.

It should finally be noted that, under this assumption, the Liouville measure dμ=dqdv, while invariant, is infinite. If, however, there exists a regular lattice of rank *d* for which we have that for every point *z* of this lattice, Q+z=Q, then we say that the corresponding Lorentz process is *periodic*. In this case, the Liouville measure is finite (more exactly, its factor with respect to the lattice is finite). (For simplicity, the lattice of periodicity will be taken as $Z^{d}$.)

**Definition** **5** (periodic Lorentz process)**.** *The Lorentz process is called* periodic *if the configuration $\left\{O_{\alpha }|1\leq \alpha <\infty \right\}$ of its scatterers is $Z^{d}$-periodic.*

**Figure 1:**
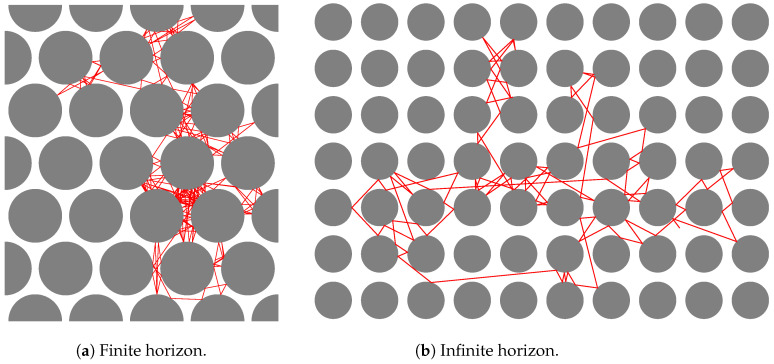
Periodic Lorentz process.

Figure 1 shows examples of trajectories (red lines) in periodic Lorentz processes (for the finite- vs. the infinite-horizon cases, respectively, cf. Definition 4).

**Definition** **6** (locally perturbed (periodic) Lorentz process)**.** *If one changes arbitrarily the scatterer configuration of a periodic Lorentz process in a bounded domain, then we talk about a* locally perturbed Lorentz process (LPLP)*. This process has a* finite or an infinite horizon *if the original periodic Lorentz process had a finite or an infinite horizon, respectively.*

#### 2.2.3. Recurrence of Stochastic Processes

**Definition** **7** (recurrence)**.** *A stochastic process in $Z^{d}$ or in $R^{d}$ (d≥1) is* recurrent *if for any bounded subset of $Z^{d}$ (or of $R^{d}$) it is true that the process returns to the subset infinitely often with probability 1.*

## 3. Recurrence of Periodic Random Walks and Lorentz Processes in the Plane

In this section, we restrict ourselves to the case when—on the one hand—the transition probabilities of the random walk are translation-invariant and—on the other hand—the Lorentz process is periodic.

### 3.1. Random Walks

Start with the classical theorem of Pólya.

**Theorem** **1** ([1])**.** *The SSRW is recurrent if d=1,2, and otherwise it is transient.*

For more general random walks, we refer to results of Chung-Ornstein and Breiman.

**Theorem** **2** ([14,15])**.**

*1.*  *Assume $\left(S_{0},S_{1},S_{2},\ldots \right)$ is a translation-invariant RW on Z. If $EX_{1}=0$, then the RW is recurrent.*
*2.*  *Assume $\left(S_{0},S_{1},S_{2},\ldots \right)$ is a translation-invariant RW on $Z^{2}$. If $EX_{1}=0$ and $E|X_{n}{|}^{2}<\infty$, then the RW is recurrent.*

### 3.2. Lorentz Processes

Based on the analogy with random walks, for periodic Lorentz processes, the exact analogue of Pólya’s theorem known for random walks had been expected.

#### 3.2.1. Finite Horizon

The first positive result was obtained in [16], where a slightly weaker form of recurrence was demonstrated: the process almost surely returns infinitely often to a moderately (actually logarithmically) increasing sequence of domains. The authors used a probabilistic method combined with the dynamical tools of Markov approximations. (The weaker form of recurrence was the consequence of the weaker form of their local limit theorem based on the weaker CLT of [17].)

An original approach appeared in 1998–99, when, independently, Schmidt [2] and Conze [3] were, indeed, able to deduce recurrence from the global central limit theorem (CLT) of [18] by adding (abstract) ergodic theoretic ideas.

**Theorem** **3** ([2,3])**.** *The planar Lorentz process with a finite horizon is almost surely recurrent.*

Their approach seems, however, to be essentially restricted to the planar finite-horizon case.

#### 3.2.2. Infinite Horizon

For attacking the infinite-horizon case, the authors of [4] returned to the probabilistic approach via the local limit theorem and first gave a new proof of the theorems of Conze and Schmidt. Finally, in [5], they could prove a local limit theorem for the Lorentz process in the infinite-horizon case that already implied recurrence in this case, too.

**Theorem** **4** ([5])**.** *The planar Lorentz process with an infinite horizon is almost surely recurrent.*

## 4. Recurrence Properties of Locally Perturbed Planar Lorentz Processes

### 4.1. Finite-Horizon Case

Answering Sinai’s 1981 question, we could prove the following:

**Theorem** **5** ([6])**.** *The locally perturbed planar Lorentz process with a finite horizon is almost surely recurrent.*

We note that the proof of the above theorem uses delicate recurrence properties of the periodic Lorentz process (cf. [7]), being interesting in themselves. Actually, they are analogues of several properties of classical random walks.

### 4.2. Infinite-Horizon Case

**Conjecture** **1.** *The locally perturbed planar Lorentz process with an infinite horizon is almost surely recurrent.*

## 5. Recurrence Properties of Symmetric vs. Locally Perturbed Random Walks with Unbounded Jumps

For understanding the difficulties in proving Conjecture 1, it should be instructive to answer the analogous question for LPRWs with unbounded jumps.

For LPRWs with bounded jumps, the first result related to Sinai’s question was given in the paper [19].

### 5.1. Reminder of Some Results of  [19]

We recall a simple example of its main theorem.

**Definition** **8.** *Let $\left\{S_{n}|n\geq 0\right\}$ be a simple RW on $Z^{2}$ such that for i=1,2*

$$P\left(X_{n+1}=\pm e_{i}|S_{n}\right)=\left\{\begin{array}{cc} \;\frac{1}{4} & \mathrm{if}\;\;S_{n}\neq \left(0,0\right)\\ \mathrm{arbitrary} & \mathrm{if}\;S_{n}=\left(0,0\right) \end{array}\right.$$

*where $e_{1}=\left(1,0\right)$ and $e_{2}=\left(0,1\right)$.*

Let, moreover,

$$U_{n}\left(t\right):=n^{-1/2}S_{\left[nt\right]}\;t\in \left[0,1\right]$$

**Theorem** **6** ([19])**.** *As n→∞*

$$U_{n}\left(t\right)\Rightarrow W\left(t\right)$$

*weakly in C[0,1], where W is the standard planar Wiener process.*

**Remark** **2.**

*1.*  *By applying the methods of [7,19], one can easily see the following:*
  *(a)*  *For $U_{n}\left(1\right)$, the global CLT holds;*
  *(b)*  *For $U_{n}\left(1\right)$, the local CLT also holds;*
  *(c)*  *As a consequence of the later one, the RW of Definition 8 is recurrent.*
*2.*  *In fact, Theorem 6 is the special case of a more general theorem of [19], whose statement roughly says that if one has a CLT for a translation-invariant RW, then changing the jump probabilities in a bounded domain does not change the statement of the CLT. The methods of [7] also imply the truth of the local limit theorem and recurrence in this generality.*

### 5.2. A Locally Perturbed RW with Unbounded Jumps

Following the bounded jumps case, for the unbounded jumps case we will also start with a simple example:

**Definition** **9.** *Let $\left\{S_{n}|n\geq 0\right\}$ be an RW (with unbounded jumps) such that for i=1,2 one has*

$$\left\{\begin{array}{cc} P(X_{n+1}=\pm e_{i}|S_{n}=\left(0,0\right)) & =1/4\\ P(X_{n+1}=\pm ne_{i}|S_{n}\neq \left(0,0\right)) & =const.\frac{1}{{\left|n\right|}^{3}} \end{array}\right.$$

Let us explain why we suggest first the study of exactly this example. The simplest example of a periodic Lorentz process is the following: all scatterers are circles of radius *R* with $R_{\min}<R<\frac{1}{2}$. This condition ensures that all elements of $M_{\mathrm{free}}$ are parallel to one of the axes. In this case, the long jumps of the Lorentz process are almost parallel to one of the axes, and the distribution of their lengths is asymptotically $const.\frac{1}{{\left|n\right|}^{3}}$ and thus belongs to the non-standard domain of attraction of the normal law (cf. [5,20,21,22]).

The unperturbed RW corresponding to the previous example belongs to the non-standard domain of attraction of the normal law with $\sqrt{n\log n}$ scaling. Denote

$$V_{n}\left(t\right):={\left(n\log n\right)}^{-1/2}S_{\left[nt\right]}\;t\in \left[0,1\right]$$

A special case of the main result of the work [23] is the following:

**Theorem** **7** ([23])**.** *As n→∞*

$$V_{n}\left(t\right)\Rightarrow CW\left(t\right)$$

*weakly in C[0,1], where W is the standard planar Wiener process and C>0.*

**Conjecture** **2.** *In the setup of this theorem, the following hold:*

*1.*  *The local version of the limit law for $V_{n}\left(1\right)$ is also true;*
*2.*  *The RW defined in Definition 9 is recurrent.*

## 6. Strongly Perturbed RWs

For locally perturbed random walks, it was sort of expected that a local perturbation should not change the limiting behavior of the RW whether the jumps are bounded or unbounded, whatever difficulties the proofs of these statements would bring up. However, under what kind of extended perturbations the classical limiting behavior of the random walk survives is intriguing. Before a precise formulation of this question, let us introduce notations.

**Definition** **10** (strongly perturbed random walks)**.** *Assume $\left\{0<a_{n}|n\geq 1\right\}$ are such that $\lim_{n\to \infty }a_{n}=\infty$. The sequence $S_{0}^{a_{n}},S_{1}^{a_{n}},S_{2}^{a_{n}},\ldots ,S_{n}^{a_{n}},\ldots$ of $a_{n}$-locally perturbed random walks is called strongly $a_{n}$-perturbed.*

**Question** **1.** *For a sequence $\left\{a_{n}|\lim_{n\to \infty }a_{n}=\infty \right\}$, denote*

$$Z_{n}\left(t\right):=n^{-1/2}S_{\left[nt\right]}^{a_{n}}\;t\in \left[0,1\right],$$

*where the jump probabilities of the translation-invariant random walk are as in Definition 8.*

*1.*  *Find a sequence $\left\{a_{n}|\lim_{n\to \infty }a_{n}=\infty \right\}$ and a strongly $a_{n}$-locally perturbed sequence of random walks (cf. Definition 2) with bounded jumps such that*
(1) $$Z_{n}\left(t\right)\Rightarrow W\left(t\right)$$
  *weakly in C[0,1].*
*2.*  *Can you prove Equation (1) for any sequences of $a_{n}$ with $a_{n}=o\left(n^{1/2}\right)$?*

**Question** **2.** *For a sequence $\left\{a_{n}|\lim_{n\to \infty }a_{n}=\infty \right\}$, denote*

$$Y_{n}\left(t\right):={\left(n\log n\right)}^{-1/2}S_{\left[nt\right]}^{a_{n}}\;t\in \left[0,1\right]$$

*where the jump probabilities of the translation-invariant random walk are as in Definition 9.*

*1.*  *Find a sequence $\left\{a_{n}|\lim_{n\to \infty }a_{n}=\infty \right\}$ and a strongly $a_{n}$-locally perturbed sequence of random walks with unbounded jumps such that*
(2) $$Y_{n}\left(t\right)\Rightarrow W\left(t\right)$$
  *weakly in C[0,1].*
*2.*  *Can you prove Equation (2) for any sequences of $a_{n}$ with $a_{n}=o\left({\left(n\log n\right)}^{1/2}\right)$?*

## Data Availability

The original contributions presented in the study are included in the article, further inquiries can be directed to the corresponding author.
